# Neutrophil lymphocyte ratio as predictor of mortality in pediatric patients with bacterial meningitis: A retrospective cohort study

**DOI:** 10.1016/j.amsu.2021.103191

**Published:** 2021-12-21

**Authors:** Hardiman Widjaja, Desy Rusmawatiningtyas, Firdian Makrufardi, Eggi Arguni

**Affiliations:** Department of Child Health, Faculty of Medicine, Public Health and Nursing, Universitas Gadjah Mada/Dr. Sardjito Hospital, Yogyakarta, 55281, Indonesia

**Keywords:** Neurtophil lymphocyte ratio, Predictor factor, Bacterial meningitis, Mortality

## Abstract

**Background:**

Bacterial meningitis causes high mortality rates among children. Even with early diagnosis and prompt treatment, around 15% of patients die especially in the first and second days after diagnosis. The neutrophil lymphocyte ratio has been reported to be a predicting factor of severity and outcome for patients with pneumonia and sepsis. However, only a few studies are available to rate the neutrophil lymphocyte ratio as a predictor of mortality in bacterial meningitis. This study aimed to know the role of the neutrophil lymphocyte ratio as a predictor of mortality in patients with bacterial meningitis.

**Methods:**

This retrospective study was conducted at Dr. Sardjito General Hospital, Yogyakarta, Indonesia between January 2016 to December 2020. Multivariate analysis was used to assess the correlation between predicting factors and outcomes using logistic regression analysis.

**Results:**

A total of 94 samples were included and analyzed in this study with bacterial meningitis. Neutrophil lymphocyte ratio >5.225 was a significant predictor of mortality in patients with bacterial meningitis with *p* = 0.004 and risk ratio 10.78. Other factors that were significant predictors of mortality included the pediatric coma scale ≤8 and positive cerebrospinal fluid culture.

**Conclusion:**

Neutrophil lymphocyte ratio is a statistically significant predictor of mortality in patients with bacterial meningitis, and can be used as a parameter to predict outcomes in patients with bacterial meningitis.

## Highlights

• Bacterial meningitis had high mortality rate among children.
• Few research was available to rate NLR.
• Neutrophil lymphocyte ratio as predictor of mortality.

## 1 Introduction

Meningitis is an acute infection of the central nervous system, involving inflammation of the lining of the brain or meninges. Meningitis is a major challenge for global health because it has high morbidity and mortality rates in developing countries [1].

Bacterial meningitis has a high mortality rate in children. Even with early diagnosis and appropriate treatment, approximately 15% of patients die, especially within 1–2 days of the onset of symptoms. In addition, patients who survive have a 10–20% chance of developing sequelae. In developing countries, the incidence of sequelae is much higher, as much as 50–65%. Globally, the three most common organisms causing bacterial meningitis are *N. meningitides*, *S. pneumoniae* and *H. influenza type b* [2].

The prognosis and outcome of bacterial meningitis are affected by many factors including age, causative bacteria, and onset of starting antibiotics. Younger age and delay in antibiotic administration lead to a poorer outcome. Loss of consciousness at the time of initial hospital admission increases the risk of death and neurological sequelae. Knowledge of these prognostic factors is important for more aggressive therapy and intensive monitoring in patients who are at risk of mortality and morbidity [3,4].

From a study in several hospitals in Yogyakarta, neurological sequelae occurred in 65% of patients with bacterial meningitis. Risk factors for neurologic sequelae in this study were seizures >30 min, pediatric coma scale (PCS) < 8 and uncontrolled seizures more than 72 h [5].

Neutrophil lymphocyte ratio (NLR) is a biomarker derived from leukocytes as a marker of inflammation. The NLR describes information from the innate and adaptive immune systems, and is a reliable parameter to describe the immune response to various stimuli/stressors. The NLR is calculated by dividing the absolute neutrophil count by the absolute lymphocyte count, and is an easy-to-use and efficient parameter [6].

In the case of bacterial meningitis, research on NLR as a predictor of mortality has not been widely studied. From a previous study by Jeppe et al., in 2020, NLR affects the prognosis of patients with bacterial meningitis. Patients with high NLR have a high cerebral blood flow velocity which are associated with a poor prognosis in patients with bacterial meningitis [6]. In Indonesia, studies of NLR as a predictor of mortality in patients with bacterial meningitis have not been widely reported until the time this study was conducted. This study is expected to explain the role of NLR as a predictor of mortality in patients with bacterial meningitis.

## 2 Methods

### 2.1 Patients and study design

This retrospective study used a cohort design among patients with bacterial meningitis. This research was conducted at Dr. Sardjito General Hospital in Yogyakarta, Indonesia. The data were collected from the medical records of patients who were treated during the period January 2016 to December 2020.

The inclusion criteria of research subjects were all pediatric patients with a diagnosis of bacterial meningitis at Dr. Sardjito Hospital for the period 2016–2020 and who have complete blood laboratory results with a neutrophil count and lymphocyte count at admission in the hospital. Exclusion criteria of study subjects were patients with immunodeficiency such as malignancy, patients with HIV, patients with long-term use of immunosuppressant drugs and patients with meningitis who did not undergo lumbar puncture. The sample size in this study was 94 subjects calculated by a formula using a 95% confidence level (CI) and 90% power.

The dependent variable in this study is mortality. The independent variables of this study are the ratio of neutrophils to lymphocytes, age (≤5 years and >5 years), nutritional status (malnutrition and adequate nutrition), duration of symptoms (≤3 days and >3 days) and the degree of consciousness when coming to the hospital (PCS ≤8 and PCS >8).

This research received approval from the Medical and Health Research Ethics Committee of the Faculty of Medicine, Public Health and Nursing, Universitas Gadjah Mada with license number KE/FK/0283/EC/2021. The study was registered at the “Research Repository Faculty of Medicine, Public Health and Nursing, Universitas Gadjah Mada” with unique identifying number UIN of 202109105. This study has been reported in line with the Strengthening the Reporting of Cohort Studies in Surgery (STROCSS) criteria [7].

### 2.2 Data analysis

Statistical analysis was performed using SPSS version 25 for Windows (IBM Corp., Armonk, NY). Bivariate analysis with Chi-square test (if there is no expected count <5) or Fisher's exact test (if there is an expected count <5) was used to analyze the relationship between predictor variables and outcomes. To determine the significance of the results of statistical calculations, a significance limit of 0.05 was used. If the *p* value was <0.25 then the independent variables were included in the multivariate analysis using the logistic regression analysis method. The predicting factor with a final value of *p* < 0.05 from multivariate analysis showed that the actual factors were significantly associated with mortality in patients with bacterial meningitis. The relationship between predictor factors and outcomes was assessed using a risk ratio (RR) with a 95% CI.

## 3 Results

During 2016 to 2020 there were 105 pediatric patients with bacterial meningitis who were treated at Dr. Sardjito Hospital. The number of samples that met the inclusion and exclusion criteria and analyzed in this study were 94 patients. Eleven patients were excluded from the analysis because they did not meet the criteria. From the results of the sample calculation, in this study the number of samples should be 47 patients in each group, but in data collection during the specified period, there were 30 patients who died in this study period and the remaining 64 patients survived.

The basic characteristics of the research subjects can be seen in Table 1. Of the total 94 patients, 52 (55%) of the patients were male. The mean age of the patients in this study was 3.91 years. The most common symptom in this study was seizures, which happened in 74 patients (78.7%), while fever occurred in 32 patients (34%), along with the symptom of loss of consciousness. Most of the patients in this study had negative culture results on both blood cultures (90.4%) and cerebrospinal (CSF) cultures (79.8%). The mean NLR value was 7.15. In this study 30 patients (31.9%) died during treatment.

**Table 1:** Subject characteristics.

		Total (N = 94)	
		N (%)	Mean(±SD)
Sex	Boy	52 (55.3)	
	Girl	42 (44.7)	
Age(Mean ± SD)			3.91 (±4.36)
Age	>5 y.o	26 (27.6)	
	≤5 y.o	68 (72.4)	
Onset of symptom(Mean ± SD)			4.67 (±3.67)
Onset of symptom	>3 days	47 (50)	
	≤3 days	47 (50)	
Nutritional status	Severe malnutrition	11 (11.7)	
	malnutrition	19 (20.2)	
	Good	59 (62.7)	
	Overweight	4 (4.2)	
	Obese	1 (1)	
Symptoms	Seizure	74 (78.7)	
	Fever	32 (34.04)	
	Decrease of consciousness	32 (34.04)	
	Vomiting	2 (2.1)	
	Headache	2 (2.1)	
PCS(Mean ± SD)			9.81 (±3.19)
PCS	>8	64 (68.1)	
	≤8	30 (31.9)	
Blood culture	Positive	9 (9.6)	
	Negative	85 (90.4)	
CSF culture	Positive	19 (20.2)	
	Negative	75 (79.8)	

PCS: Pediatrics coma scale, CSF: Cerebrospinal fluid, SD: Standard deviation.

The results of blood and CSF cultures in this study are shown in Table 2. From the results of cultures, blood cultures only grew in 9 patients (9.6%) with the most bacteria being *Staphylococcus aureus*, *Staphylococcus hominis* and *Pseudomonas aeruginosa*, two patients each. For CSF culture, bacteria growth was found in 19 patients (20.6%). The most common bacteria that grew were *Staphylococcus haemolyticus* and *Staphylococcus hominis* in four patients (21%).

**Table 2:** Blood and CSF culture.

Microorganism	Dead n(%)	Alive n(%)
Blood culture (N = 9)		
*Staphylococcus aureus*	1(50)	1(50)
*Pseudomonas aeroginosa*	–	2(100)
*Staphylococcus hominis*	2(100)	–
*Staphylococcus capitis*	1(100)	–
*Streptococcus pneumoniae*	1(100)	–
*Staphylococcus warneri*	1(100)	–
CSF culture (N = 19)		
*Staphylococcus haemolyticus*	2(50)	2(50)
*Staphylococcus epidermidis*	3(75)	1(25)
*Salmonella* sp	1(33.3)	2(66.7)
*Staphylococcus aureus*	2(100)	–
*Staphylococcus capitis*	–	1(100)
*Staphylococcus hominis*	–	1(100)
*Escherichia coli*	–	1(100)
*Enterococcus faecium*	1(100)	–
*Streptococcus pneumoniae*	1(100)	–
*Pantoea* sp	–	1(100)

CSF: Cerebrospinal fluid.

**Fig. 1:**
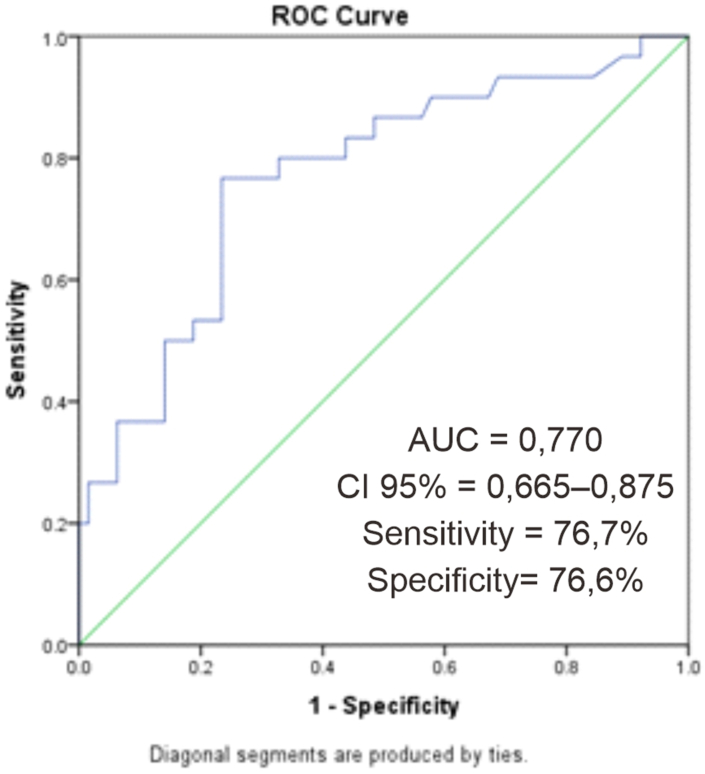
The ROC curve of predictor of mortality in pediatric bacterial meningitis patients.

In this study, the optimal cut-off for NLR as a predictor of mortality in patients with bacterial meningitis was calculated. Using the receiver operating characteristic (ROC) curve, the optimal cut off result is 5.225. By using a cutoff point of 5.225, the NLR has a sensitivity of 76.7% and a specificity of 76.6%. NLR was statistically significant as a predictor of mortality in bacterial meningitis patients with *p* < 0.001 and RR 4.84 (Fig. 1).

From the bivariate analysis using the Chi square test shown in Table 3, the factors that were statistically significant (*p* < 0.05) as predictors of mortality in pediatric patients with bacterial meningitis were NLR >5.225 (RR 4.84; 95% CI: 2.31–10.14; *p* < 0.001), PCS value 8 (RR 0.31; 95% CI: 0.17–0.56; *p* < 0.001), positive blood culture (RR 2.36; 95% CI: 1.33–4.19; p = 0.027 and positive CSF culture (RR 1.97; 95% CI: 1.12–3.48; *p* = 0.030). In this study, there were no statistically significant differences between gender, age, duration of symptoms and nutritional status.

**Table 3:** Bivariate and multivariate analysis of NLR and other predictors of child with bacterial meningitis.

Parameters	Outcome		Bivariate analysis		Multivariate analysis	
	Died N(%)	Survived N(%)	*P*	RR (CI 95%)	*P*	RR(CI 95%)
NLR			0.000	4.84 (2.31 – 10.14)	0.004	10.78 (3.23 – 35.96)
>5.225	23 (60.5%)	15 (39.5%)				
≤5.225	7 (12.5%)	49 (87.5%)				
Sex			0.110	0.62 (0.34–1.12)	0.206	0. 46 (0.14–1.53)
Boy	13 (25.0%)	39 (75%)				
Girl	17 (40.5%)	25 (59.5%)				
Age			0.445	1.29 (0.65–2.55)		
>5 years old	8 (30.8%)	22 (69.2%)				
≤5 years old	22 (32.3%)	42 (67.6%)	0.658	0.88 (0.48–1.58)		
Onset of symptoms						
>3 days	14 (29.8%)	33 (70.2%)				
≤3 days	16 (34%)	31 (66%)				
Nutritional status malnutrition	4 (36.3%)	7 (63.7%)	0.721	1.29 (0.57–2.95)		
good nutrition	26 (31.3%)	57 (68.7%)				
PCS			0.000	0.31 (0.17–0.56)	0.004	0.17 (0.05–0.58)
>8	12 (18.8%)	52 (81.3%)				
≤8	18 (60.0%)	12 (40%)				
Blood culture			0.027	2.36 (1.33–4.19)	0.153	4.19 (0.58–30.01)
Positive	6 (66.7%)	3 (33.3%)				
Negative	24 (28.2%)	61 (71.8%)				
CSF culture			0.030	1.97 (1.12–3.48)	0.035	4,54 (1.11–18.54)
Positive	10 (5.6%)	9 (47.4%)				
Negative	20 (26.7%)	55 (73.3%)				

NLR: Neutrophil lymphocyte ratio, PCS: Pediatrics coma scale, CSF: Cerebrospinal fluid, RR: Relative risk, CI: Confidence interval.

Based on the bivariate analysis, variables with *p* value < 0.25 were continued in multivariate analysis, namely NLR, gender, PCS, blood culture and CSF culture. From the multivariate logistics regression analysis, only NLR (RR 10.78; 95% CI: 3.23–35.96; *p* = 0.0041), PCS (RR 0.17; 95% CI: 0.05–0.58; *p* = 0.004) and CSF culture (RR 4.54; 95% CI: 1.11–18.54; *p* = 0.035) were significantly correlated with for the outcome of mortality in pediatric patients with bacterial meningitis, while the blood culture and gender were not statistically significant (*p* > 0.05).

## 4 Discussion

Acute bacterial meningitis remains a challenge for global health with high mortality and morbidity despite advances in antibiotic therapy and modern vaccine strategies. Children are especially more susceptible to bacterial meningitis because their immune systems are not fully developed. The World Health Organization (WHO) estimates that every year there are 170,000 deaths due to bacterial meningitis, and the case fatality rate can be up to 50% if adequate treatment is not given [8].

From this study, it was found that patients with NLR values above 5.225 died more (60.5%) than patients with NLR 5.225 or less (12.5%), with RR 4.84. High NLR values in patients with bacterial meningitis are associated with increased mortality rates. From a study by Sharew et al., sepsis is one of the most common causes of death in patients with bacterial meningitis (39%) [9]. This NLR correlation with the occurrence of sepsis was also found in a study by Zahorec which indicated a decrease in the number of neutrophils and an increase in the number of lymphocytes were needed to improve the clinical condition of patients with SIRS and sepsis [10].

Several studies have shown impaired neutrophil function during sepsis. Neutrophils play a major role in the natural defense against infection by eliminating pathogens. Neutrophils are not only associated with increased respiratory burst capacity but also decreased apoptosis. The presence of delayed apoptosis in inflammatory conditions will inhibit the killing ability of neutrophil cells and their anti-inflammatory role, resulting in SIRS and MOD/F [10].

Decreased consciousness on admission at the hospital is a predictor of poor outcome in patients with bacterial meningitis. Approximately 15–20% of patients with bacterial meningitis have decreased consciousness when they first come to the hospital. This condition is associated with high rates of morbidity and mortality. In one study, only 1 in 5 patients (20%) survived [11].

Malnutrition is a risk factor for mortality in children with bacterial meningitis [12]. In this study, nutritional status did not significantly affect mortality in children with bacterial meningitis (*p* = 0.785). In this study, most of the patients were well-nourished (68.2%) and poor nutrition was only found in 11 patients (11.7%). Malnutrition can make children more susceptible to infection. Infection can also contribute to malnutrition. This creates a vicious cycle that is difficult to overcome. Protein energy malnutrition significantly increases the morbidity and mortality of children with bacterial meningitis, although the mechanism by which this occurs is still unclear [13,14].

This current study reported 9.6% of positive blood and 20.6% of CFS cultures. The low rate of positive result might be caused by administration of intravenous antibiotics prior to the blood and lumbar puncture [5]. Of the 19 patients with positive CSF cultures, 10 patients (52.6%) died. The most common germs obtained from CSF culture were *Staphylococcus haemolyticus* and *Staphylococcus epidermidis*, each found in four patients. Research by Migrovic et al., in 2008 revealed that administration of antibiotics before a lumbar puncture did not affect the leukocyte count in the CSF, but the administration of antibiotics before the lumbar puncture was associated with increased glucose and decreased protein in the CSF and significantly reduced the positivity rate of CSF culture. This effect began to be seen after 4 h of antibiotic administration, but a significant difference in CSF profile occurred after 12 h of antibiotic administration [15,16].

In this study, the optimal cutoff value for NLR was calculated. By using the ROC curve, the optimal cut off value for NLR is 5.225. The cutoff value for NLR is different in each study depending on the type of disease being studied. In a study conducted by Forget et al., the normal range of NLR values in a healthy population was 0.78–3.581 [5]. In another study by Gurol et al. in patients with sepsis, the NLR value in local infections was 5.68 and in systemic infections, it was 11.78–13.16 [17].

Research on NLR as an outcome prognostic factor in cardiovascular disease, malignancy and infection has been widely done, but studies of cases of bacterial meningitis are still rarely done, especially in Indonesia. We are aware of the small sample size as one of the limitations of this study. The lack of comorbidities data may also affect NLR values and influence the outcomes of patients with bacterial meningitis. Further studies with larger sample sizes are needed to confirm these findings.

## 5 Conclusions

Pediatric patients with bacterial meningitis and high NLR values need closer monitoring and may require more aggressive management in order to improve patient outcomes. Other studies that include any comorbidities present in patients and are conducted with a larger sample size are needed to validate the effect of NLR values on the outcome of patients with bacterial meningitis.

## Provenance and peer review

Not commissioned, externally peer reviewed.

## Sources of funding for your research

The authors declare that this study had no funding source.

## Ethical approval

This study has been approved by the Ethical Committee of Faculty of Medicine, Public Health and Nursing, Universitas Gadjah Mada/Dr. Sardjito Hospital (Ref: KE/FK/0283/EC/2021)

## Consent

Written informed consent was obtained from the patient for publication of this study and accompanying images. A copy of the written consent is available for review by the Editor-in-Chief of this journal on request.

## Author contribution

Hardiman Widjaja, Desy Rusmawatiningtyas, and Eggi Arguni conceived the study, drafted the manuscript, and critically revised the manuscript for important intellectual content. Firdian Makrufardi drafted the manuscript and critically revised the manuscript for important intellectual content. All authors read and approved the final draft. All authors facilitated all project-related tasks.

## Registration of research studies

Research Repository Faculty of Medicine, Public Health and Nursing, Universitas Gadjah Mada.

Register Unique Identifying Number (UIN): 202109105.

## Guarantor

Eggi Arguni.

## Declaration of competing interest

No potential conflict of interest relevant to this article was reported.
